# Investigation on Mechanical and Fatigue Performance of Large-Thickness Flexible Base Layer Asphalt Pavement

**DOI:** 10.3390/ma19071446

**Published:** 2026-04-04

**Authors:** Yihua Nie, Shuaihua Wang, Ruoxi Zhang, Bo He, Guosen Yao, Long Chen

**Affiliations:** 1 School of Civil Engineering, Hunan University of Science and Technology, Xiangtan 411201, China; 19854811213@163.com (S.W.); zrxjianying@163.com (R.Z.); 19138350363@163.com (B.H.); 13502225937@163.com (G.Y.); 2 Hunan University of Science and Technology Engineering Testing Co., Ltd., Xiangtan 411201, China; 3 School of Civil and Environmental Engineering, Hunan University of Science and Engineering, Yongzhou 425199, China

**Keywords:** asphalt pavement, large thickness flexible base layer, mechanical property, fatigue characteristic, long-life judgment

## Abstract

A static load test, single-wheel load test, and cyclic-wheel load test were carried out on large-thickness flexible base-layer and semi-rigid base-layer asphalt pavement structures by a multifunctional wheel-load testing machine. A comparative analysis was conducted on the influence and mechanism factors, such as load strength, test temperature, and load rate, on the stress and strain at the top and bottom of two asphalt pavement structures. The results show that in the interval of 1.3 MPa ≥ load intensity ≥ 0.5 MPa, with the increase of static load, the transverse strain and vertical strain at the top and bottom of the base layer of large-thickness flexible base-layer asphalt pavements increase slowly with a slight increase; the transverse strain and vertical strain at the top of the base layer of large-thickness semi-rigid base-layer asphalt pavements are more sensitive to heavy traffic load; and the transverse strain and vertical strain generated at the bottom of the base layer increase uniformly with the enhancement of static load. Under the action of a single-wheel load, the transverse and vertical strain generated at the top and bottom of the base layer of large-thickness flexible base-layer and semi-rigid base-layer asphalt pavements are alternately tensile and compressive, mainly compressive strains, while large-thickness semi-rigid base-layer asphalt pavement exhibits more complex strain changes.

## 1. Introduction

In recent years, with the rapid development of the transportation industry, road traffic load has increased, which means that asphalt pavement materials face more severe environmental and higher use requirements, in which the mechanical and fatigue properties of base-layer materials have gradually become a key issue in road construction and maintenance. Cement-stabilized aggregates are commonly used as a semi-rigid base layer of asphalt pavement due to their good slab properties and high bearing capacity; however, they are prone to induce early asphalt pavement disease due to their shortcomings, such as shrinkage and cracking, poor water penetration, and sensitivity to heavy traffic, which results in the existing asphalt pavements needing frequent maintenance and repairs and a large amount of wasted resources. Based on this, road researchers have proposed the concept of durable asphalt pavement design, where the service life of asphalt pavement reaches more than 40 years without significant repairs [1,2]. Asphalt-stabilized crushed-stone mixtures have the advantages of high shear strength, good fatigue resistance, and can effectively inhibit reflective cracking, etc. Related studies have shown that the use of asphalt-stabilized crushed-stone mixtures with a flexible base layer is an effective way to achieve durable asphalt pavements [3,4,5]. In this paper, the mechanical and fatigue characteristics of large-thickness flexible base-layer and semi-rigid base-layer asphalt pavements under static and dynamic loads are compared through experimental and theoretical analyses, and the long-life judgment is analyzed, which can provide a scientific basis for the design and construction of durable asphalt pavement.

As the load-bearing layer of asphalt pavement, the mechanical and fatigue performance of the base layer is a key indicator that affects the service life and service quality of asphalt pavement [6,7,8,9]. Road researchers have conducted numerous studies on the mechanical response and fatigue performance of the base layer of asphalt pavement under loading. Assogba et al. analyzed the mechanical response of the base layer of semi-rigid base-layer asphalt pavement under the combined effects of nonlinear temperature and traffic load through three-dimensional modeling and outdoor loading test, and the results showed that the main factors affecting the mechanical properties of a semi-rigid base layer are vehicle speed and axle load amplitude [10]. Cyriaque et al. analyzed the effects of vehicle speed and overload on the dynamic response of semi-rigid base-layer asphalt pavement through transient dynamic modeling of semi-rigid base-layer asphalt pavement, and the results showed that, under the action of heavy loads, the vertical strain generated at the top of the semi-rigid asphalt pavement base has both compressive and tensile components [11]. Guo et al. analyzed the effects of heavy traffic on the mechanical properties of flexible base-layer and semi-rigid base-layer asphalt pavements by comparing field tests, and the results showed that the temperature stability and fatigue performance of flexible base-layer asphalt pavement under heavy loads were better than that of semi-rigid base-layer asphalt pavement [12,13]. Rinya et al. carried out a finite element simulation analysis on the mechanical response of road surfaces under the action of traditional virgin aggregate and a flexible base layer with recycled aggregate under load. They studied the fatigue performance of two types of base-layer asphalt pavement, and the results showed that the fatigue performance of asphalt pavement was improved by 55.81% after the replacement of traditional aggregate with recycled aggregate [14]. Coelho et al. investigated the mechanical behavior of a flexible base layer of emulsified asphalt with a mixing amount of 3% in different maintenance ages through a triaxial test, and the research showed that the design of emulsified flexible-base layer asphalt pavement must consider the plastic deformation behavior of the asphalt layer bottom [15]. Liu et al. analyzed the strain waveform of flexible base-layer asphalt pavement under different axle weights and temperatures through on-site loading tests, and the results of the study showed that the axle weights affect the vertical and transversal strain extremes of the asphalt layer bottom to a significantly different extent under different temperatures [16]. Zhuang et al. analyzed the fatigue characteristics of the asphalt layer of flexible base-layer asphalt pavement through a cyclic-wheel load test. They found that under the repeated action of wheel load, the strain of the asphalt layer bottom goes through the process of compression, then tension, finally stabilization, and the change in the strain of the asphalt layer bottom and the slope of the pavement temperature can be used as the basis for judgment of fatigue cracking in flexible base-layer asphalt pavement [17,18,19]. Jiang et al. analyzed the influence of bonding conditions on the mechanical properties of the top of flexible base-layer asphalt pavement under vertical loads through finite element simulation. The study showed that compared with the non-uniformity of wheel load, bonding conditions significantly affect the structural stability of the top of the flexible base-layer asphalt pavement [20].

Currently, most of the research on the mechanics and fatigue of flexible base-layer asphalt pavement focuses on the stress and strain responses of the road surface and the bottom of the base layer under the action of load. However, the research neglects analysis of the mechanical response of the top of the base layer under the action of load, where the top of the base layer is connected to the surface layer, and the mechanical and fatigue performance is directly related to the service life of the flexible base-layer asphalt pavement. Previous studies have typically used a single-cycle wheel-load mode to simulate and analyze the mechanical and fatigue performance of flexible-base asphalt pavement in daily use, but have overlooked the use of flexible-base asphalt pavement under special traffic conditions, such as parking and low traffic flow. Therefore, corresponding experimental simulations are needed to further investigate the mechanical and fatigue performance of flexible-base asphalt pavement under special working conditions.

Based on the above, this paper adopts static load testing (simulate parking conditions), single-wheel load testing (simulate low traffic flow conditions), and cyclic-wheel load testing (simulate daily service conditions), where the mechanical and fatigue characteristics of semi-rigid-base asphalt and flexible-base asphalt pavements’ tops and bottoms under load were compared and analyzed in a laboratory environment, as well as analyses of long-life judgment, to provide a relevant reference for the design of durable asphalt pavements.

## 2. Materials and Test Programs

### 2.1. Test Materials

In this paper, the binder selected is SBS-modified asphalt, with reference to JTG E20-2011 [21] “Test Specification for Asphalt and Asphalt Mixture in Highway Engineering”, and the relevant test requirements for its detection, its basic technical indicators, and test results are shown in Table 1.

The coarse aggregate used in the test model is limestone crushed stone with a grain size of 19–31.5 mm, 4.75–19 mm, 4.75–13.2 mm, and 2.36–9.5 mm. The fine aggregate is limestone mechanism sand with a grain size of 0–2.36 mm, and the filler is limestone mineral powder. Based on JTG E42-2005 [22] “Aggregate Testing Procedures for Highway Engineering”, the relevant test requirements for its detection, its basic technical indicators, and test results are shown in Table 2, Table 3 and Table 4.

The cement is ordinary silicate cement, which was tested with reference to the relevant test requirements of JTG 3420-2020 [23] “Test Procedures for Cement and Cement Concrete in Highway Engineering”, and its basic technical indexes and test results are shown in Table 5.

### 2.2. Sample Production

**Figure 1:**
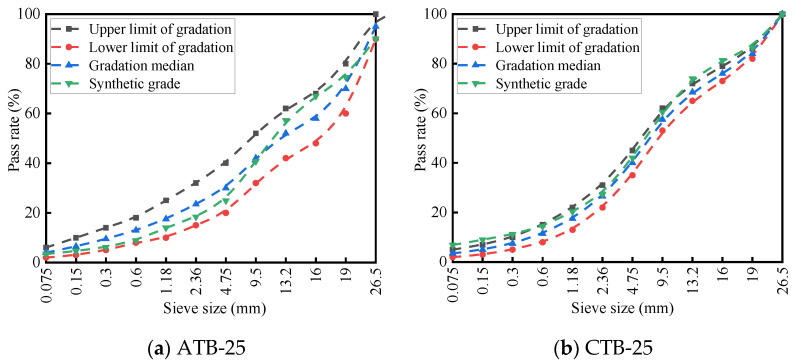
Gradation curves of the base layer.

To study the structural mechanics and fatigue performance of large-thickness flexible-base asphalt pavements, this paper introduces semi-rigid-base asphalt pavements as a comparative test. The thickness of the structural layer of the pavement is 38 cm, in which the large-thickness flexible-base asphalt pavements are constructed with an asphalt stabilized crushed-base structure (referred to as ATB pavement structure), and the semi-rigid-base asphalt pavements are constructed with a 4% cement-stabilized crushed-base structure (referred to as CTB pavement). Both types of asphalt pavements are designed with reference to JTG F40-2004 [24] “Technical Specification for Highway Asphalt Pavement Construction”, in which the grading curves of ATB flexible and CTB semi-rigid base layers are shown in Figure 1.

**Figure 2:**
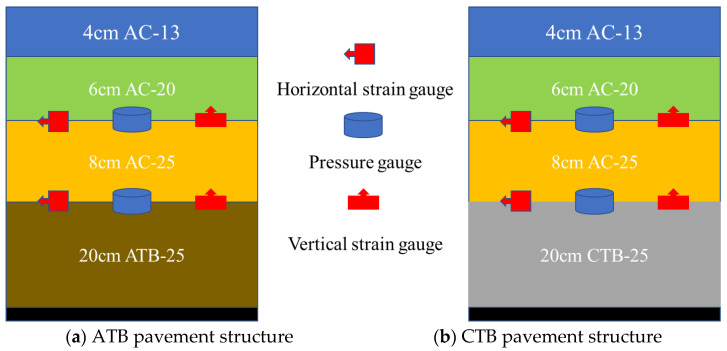
Schematic diagram of pavement structure and sensor layout.

The sensors were embedded in the specimen during the specimen preparation process, and the embedding position was determined based on the numerical simulation results to avoid the sensors from interfering with the stress and strain distribution of the specimen as much as possible. The distribution of each layer of the two types of asphalt pavements and the location of sensor burial are shown in Figure 2. The test results of the technical indicators and parameters related to ATB-25 and CTB-25 are shown in Table 6 and Table 7.

**Figure 3:**
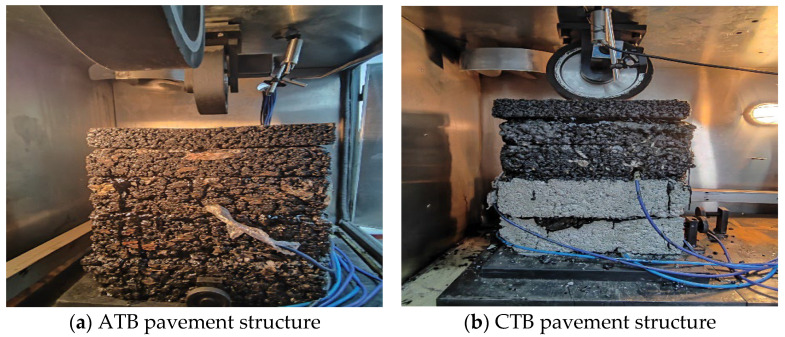
Test sample.

According to JTG E20-2011 [21] “Test Specification for Asphalt and Asphalt Mixture in Highway Engineering”, the wheel load test specimen in this paper adopts the rutted plate specimen, referring to the rutting test of asphalt mixtures, based on considerations of laboratory testing conditions and the repeatability of test results, where the specimen size is length (300 mm) × width (300 mm), and at least three samples of the same pavement structure were tested in parallel. When the coefficient of variation in the test results of the three samples is not greater than 20%, the average value shall be taken as the experimental result. support molded specimen in the reserved position into the stress and strain transducer. Modified emulsified asphalt has good workability and bonding performance in laboratory operations, which can ensure the uniformity of interlayer bonding and reduce test errors caused by uneven bonding. The layers are bonded by modified emulsified asphalt, and the molded test specimen is shown in Figure 3.

### 2.3. Test Program

**Figure 4:**
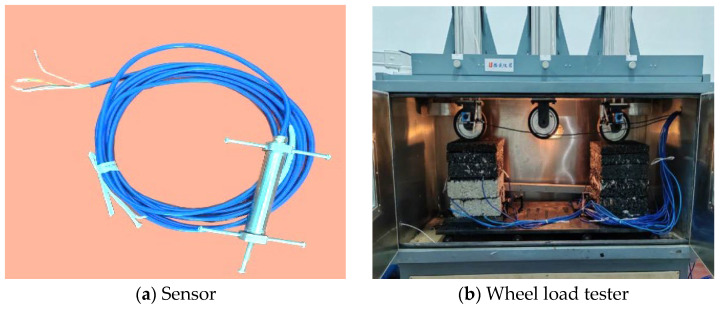
Test equipment.

This paper adopts a multifunctional wheel-load testing machine to carry out static load tests, single-wheel load tests, and cyclic-wheel load tests on large-thickness flexible base-layer and semi-rigid base-layer asphalt pavements; the related test equipment is shown in Figure 4. Stress and strain responses of the two asphalt pavement structures under different loading intensities, temperatures, and loading rates are used to study the mechanical and fatigue characteristics of asphalt pavements with large-thickness flexible base layers and analyze long-life determination. In this paper, the tensile stress and strain generated during the tests are positive, and the compressive stress and strain generated during the tests are negative.

1. Static load test

The prepared ATB and CTB specimens are maintained at normal temperature for 24 h. The load strengths were 0.5 MPa, 0.7 MPa, 0.9 MPa, 1.1 MPa, and 1.3 MPa, and the test temperature was 30 °C. The loads were loaded from the small loads to the large loads in sequence, and the loads were slowly increased to the corresponding load strengths each time. The data were collected after the loads were stabilized for 1 min. To avoid any impact on the lower-level load testing, the next level of load test was carried out after an interval of 5 min. Through the static load test, the stress and strain response characteristics of ATB and CTB asphalt pavement structures at the top and bottom of the base layer under different loads were analyzed.

2. Single wheel load test

The prepared ATB and CTB specimens were maintained at the test temperature; the single-wheel load test temperatures were 30 °C and 60 °C, the maintenance time was 24 h, and the loading strength was 0.7 MPa. The tire size of the wheel carrier had an outer diameter of 200 mm, a wheel width of 50 mm, and the wheel load rate was 21 times/min and 42 times/min. The single-wheel load test analyzes the stress and strain response characteristics of the top and bottom of the base layer of the asphalt pavement structure of ATB and CTB under different temperatures and loading rates.

3. Cyclic wheel load test

The prepared ATB and CTB specimens were maintained at the test temperature, where the cyclic loading test temperature was 30 °C, the maintenance time was 24 h, and the loading intensity was 0.7 MPa. The tire size of the wheel carrier had an outer diameter of 200 mm, a wheel width of 50 mm, and the wheel loading rate was 42 times/min. The cyclic loading test was carried out on the two types of asphalt pavement structures, and the load was applied until the destruction of the specimen or, alternatively, the number of cyclic loads reached 1 million times. The data of the whole loading process were collected, each cycle of 100,000 times was regarded as a node, and the mechanical index of the node was taken as the representative value. Through the cyclic loading test, the stress and strain response characteristics of the ATB and CTB asphalt pavement structures at the top and bottom of the base layer under cyclic loading were analyzed to ascertain the fatigue performance of asphalt pavements with a large-thickness flexible base layer and long-life judgment.

## 3. Test Results and Analysis

### 3.1. Static Load Test

**Figure 5:**
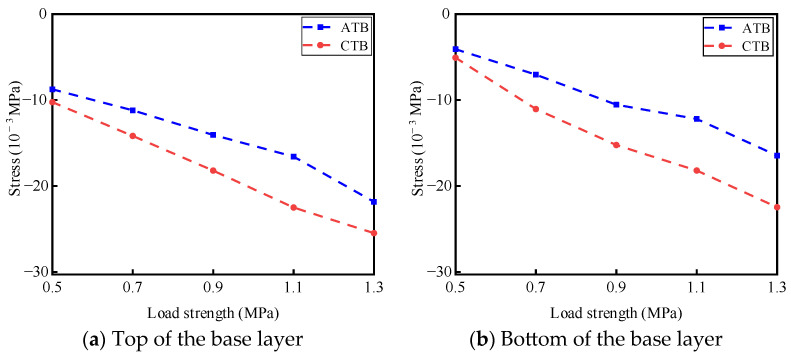
Effect of load intensity on stress.

**Figure 6:**
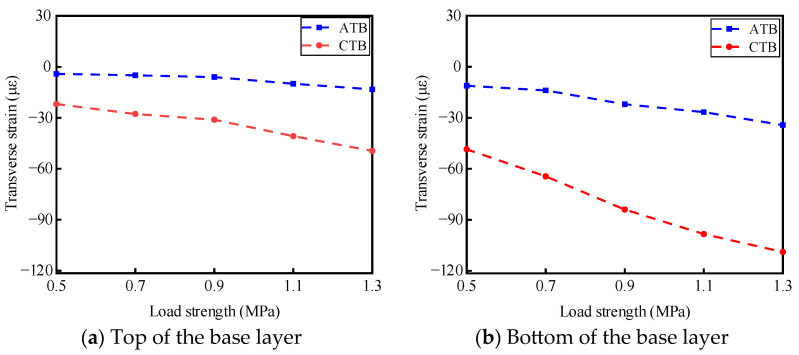
Effect of load intensity on transverse strain.

**Figure 7:**
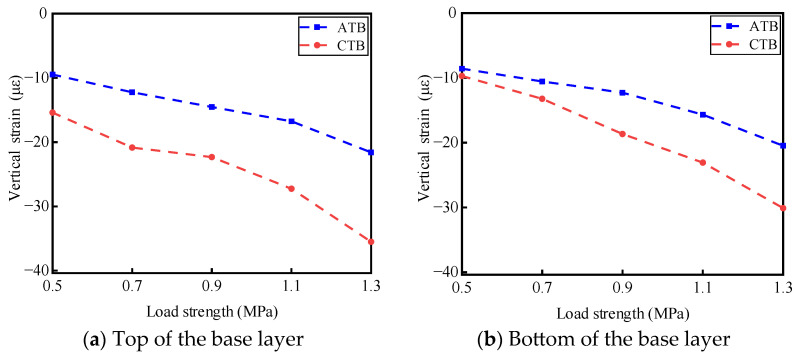
Effect of load intensity on vertical strain.

Static load tests were conducted on ATB and CTB asphalt pavement structures under different load intensities of 0.5 MPa, 0.7 MPa, 0.9 MPa, 1.1 MPa, and 1.3 MPa, and the test temperature was 30 °C. Under different load intensities, the results of the stress response of the base layer of the two types of asphalt pavement structures at the top and bottom of the base layer are shown in Figure 5. The results of the transverse strain response are shown in Figure 6. The vertical strain response results are shown in Figure 7.

#### 3.1.1. Analysis of the Law of Load Intensity Effect on Stress

As shown in Figure 5, with the enhancement of loading, the compressive stresses at the top and bottom of the base layer of large-thickness flexible base-layer asphalt pavement and semi-rigid base-layer asphalt pavement increase, and under the same loading, the compressive stresses at the top and bottom of the base layer of semi-rigid base-layer asphalt pavement are more significant than those of the flexible base-layer asphalt pavement. When the load intensity is 1.1 MPa, the difference between the compressive stresses at the top of the base layer of the two asphalt pavement structures reaches an extreme value of 6.0 × 10^−3^ MPa. When the load intensity increases from 0.9 MPa to 1.1 MPa, the trend of the compressive stress generated at the bottom of the base layer of the two asphalt pavement structures, increasing with the enhancement of load effect, becomes slower.

This is mainly due to the two types of asphalt pavement structure of the base-layer material characteristics. There are apparent differences: semi-rigid base-layer material consists of cement-stabilized gravel, with high strength and stiffness, and the flexible base layer consists of graded crushed stone, with lower stiffness and higher flexibility and the void rate is more large. Under static load, compared with a semi-rigid base layer, a flexible base layer can better absorb and disperse the stress generated by the load, and at the top and bottom of the base layer, it generates less compressive stress, reducing the stress level of the structural surface layer of asphalt pavements, which can effectively enhance the durability of the surface layer structure.

#### 3.1.2. Analysis of the Law of Influence of Load Intensity on Transverse Strain

As shown in Figure 6, with the enhancement of loading, the transverse strains at the top and bottom of the base layer of large-thickness flexible base-layer asphalt pavements and semi-rigid base-layer asphalt pavements are compressive strains, and the transverse strains increase with the enhancement of loading. Under the same load, the transverse strains at the top and bottom of the base layer for semi-rigid-base asphalt pavement structures are larger than those for flexible base asphalt pavement structures. For flexible base-layer asphalt pavements, the transverse strains at the top and bottom of the base layer increase slowly with the increase of loading, and the overall increase is slight. For semi-rigid base asphalt pavement, when the load intensity increases from 0.9 MPa to 1.3 MPa, the transverse strain at the top of the base layer increases in magnitude with the strengthening of the load and is more sensitive to the heavy traffic; the transverse strain at the bottom of the base layer increases uniformly with the strengthening of the load.

This is mainly due to the significant difference in the stress mechanism of the base layer of the two asphalt pavement structures under static load, where the semi-rigid base-layer force mechanism mainly relies on the strength and stiffness of the material to resist the load effect. The flexible base layer, through the flow of the material and re-arrangement to reduce the production of transverse strains, effectively mitigates the development of asphalt pavement surface-layer reflective cracks, prolonging its service life.

#### 3.1.3. Analysis of the Law of Influence of Load Intensity on Vertical Strain

As shown in Figure 7, with the enhancement of loading, the vertical strains at the top and bottom of the base layer of large-thickness flexible base-layer asphalt and semi-rigid base layer asphalt pavements are compressive strains. The vertical strains at the top and bottom of the base layer of semi-rigid base-layer asphalt pavements are more large than those of flexible base-layer asphalt pavement structures under the same loading. For flexible base-layer asphalt pavement, the vertical strain at the top and bottom of the base layer increases uniformly with the enhancement of loading, and the increase is slight. For semi-rigid base-layer asphalt pavement, when the load is increased from 0.7 MPa to 0.9 MPa, the enhancement of the load effect on the vertical strain at the top of the base layer has less impact. When the load exceeds 0.9 MPa, the enhancement of the load effect makes the vertical strain at the top of the base layer increase rapidly, and it is more sensitive to heavy traffic. With the enhancement of loading, the vertical strain generated at the bottom of the base layer increases uniformly.

This is mainly due to the significant differences in the structural response of the base materials of the two asphalt pavement structures under static load, where the flexible base layer has a better deformation ability and adaptability to the static load effect through the deformation of the material to reduce the transfer and accumulation of stress. This deformation ability helps to reduce the generation of vertical strains and enhance the durability of the asphalt pavement [25,26]. The semi-rigid base layer, due to its significant stiffness, responds to load more directly and violently and is prone to large stress concentration and deformation, resulting in sizeable vertical strain generated under the same load. This adversely affects the durability of asphalt pavement in the long-term service process.

### 3.2. Single-Wheel Load Test

**Figure 8:**
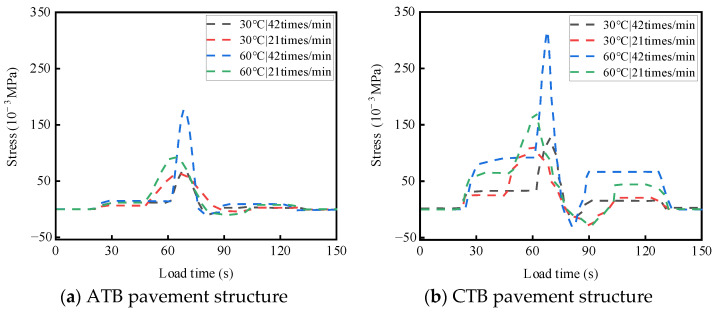
Effects of temperature and load rate on the stress at the top of the base layer.

**Figure 9:**
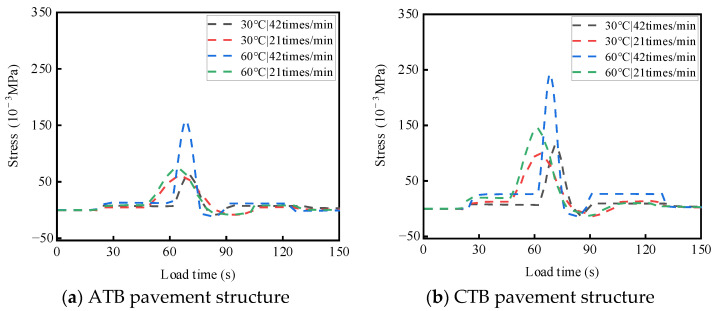
Effects of temperature and load rate on the stress at the bottom of the base layer.

**Figure 10:**
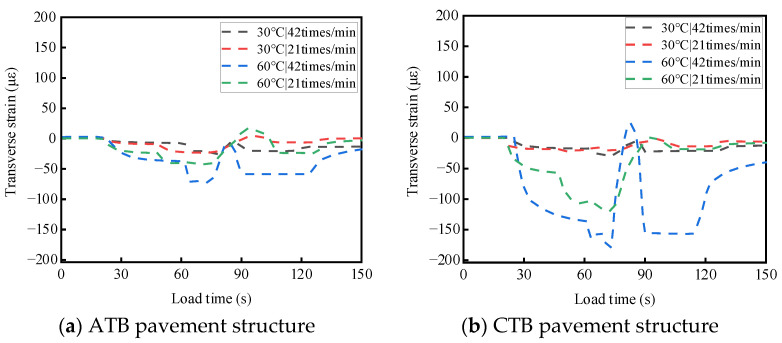
Effects of temperature and load rate on the transverse strain at the top of the base layer.

**Figure 11:**
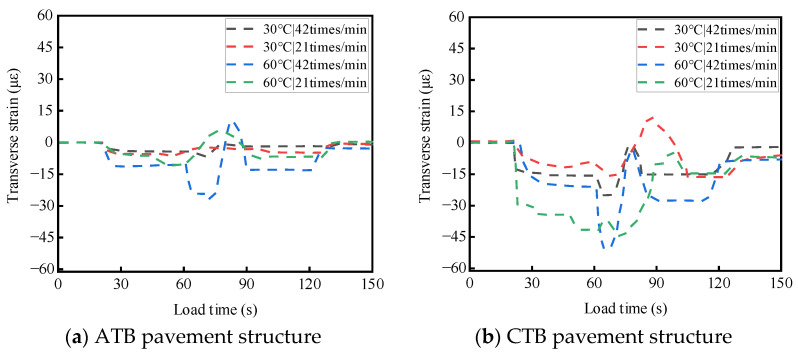
Effects of temperature and load rate on the transverse strain at the bottom of the base layer.

**Figure 12:**
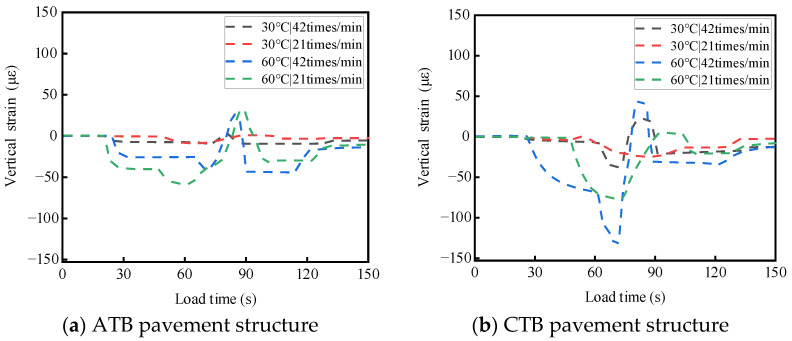
Effects of temperature and load rate on the vertical strain at the top of the base layer.

**Figure 13:**
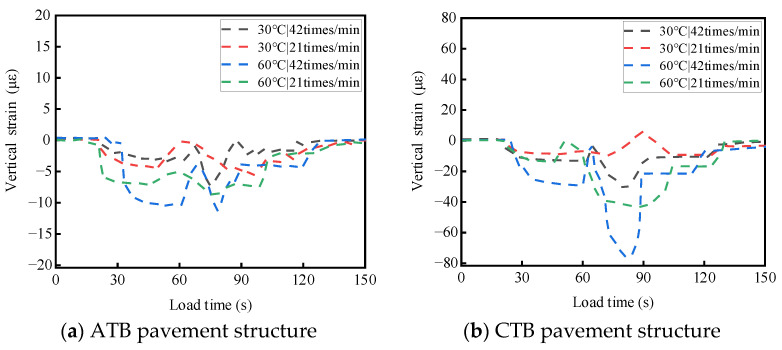
Effects of temperature and load rate on the vertical strain at the bottom of the base layer.

ATB and CTB asphalt pavements were tested at different temperatures and load rates. For a single-wheel load test, the test temperatures were 30 °C and 60 °C, the wheel load rates were 21 times/min and 42 times/min, and the load strength was 0.7 MPa; the two asphalt pavement structures of the base layers of the top and bottom of the stress response test results are shown in Figure 8 and Figure 9. The results of the transverse strain response are shown in Figure 10 and Figure 11. The vertical strain response results are shown in Figure 12 and Figure 13.

#### 3.2.1. Analysis of Temperature and Load Rate on Stress

As shown in Figure 8 and Figure 9, under the action of a single-wheel load, large-thickness flexible base-layer asphalt pavement and semi-rigid base-layer asphalt pavement, compressive stress and tensile stress are alternately at the top and bottom of the base layer, which are mainly tensile stresses. When the test temperature and loading rate are the same, compared with flexible base asphalt pavement, semi-rigid base asphalt pavement produces more complicated stress changes at the top and bottom of the base.

As shown in Figure 8a and Figure 9a, for the flexible base-layer asphalt pavement, at normal temperature (30 °C), the increase in load rate has less influence on the stress at the top and bottom of the base layer, and the flexible base layer can adapt to the changing load rate better; at a high temperature (60 °C), stress at the top and bottom of the base layer will increase with the acceleration of the load rate due to the flexible base layer having enough time to undergo viscoelastic deformation. This slow deformation makes the stress distribution inside the base material relatively uniform [27,28,29], and the stress peaks at the top and bottom of the base are relatively small.

As shown in Figure 8b and Figure 9b, for the semi-rigid base-layer asphalt pavement, at normal temperature (30 °C), the increase in load rate on the top and bottom of the base layer of the impact of stress is relatively tiny; the semi-rigid base layer can adapt to the change in load rate better. At a high temperature (60 °C), the increase in load rate makes the top and bottom base layers’ generated stresses increase rapidly, and the top of the base layer’s stress is generated, where the response is more violent, and the stress increase is more significant. This is due to the high-speed load that makes the compressive stress at the top of the base layer exceed the ultimate compressive strength of the semi-rigid base-layer material, resulting in cracks or damage at the top of the base layer, which affects the structural stability of the pavement.

#### 3.2.2. Analysis of the Temperature and Load Rate on Transverse Strain

As shown in Figure 10 and Figure 11, under the action of a single-wheel load, the transverse strains generated by large-thickness flexible and semi-rigid base asphalt pavements at the top and the bottom of the base layer are tensile and compressive alternating phenomena, mainly compressive strains. When the test temperature and loading rate are the same, the transverse strains at the top and bottom of the semi-rigid base-layer asphalt pavement are more complicated than those of the flexible base-layer asphalt pavement.

As shown in Figure 10a and Figure 11a, for flexible base-layer asphalt pavement, at a normal temperature (30 °C), the flexible base-layer material has good deformation ability and adaptability, and the increase in load rate has a relatively balanced influence on the transverse strains at the top and bottom of the base layer. At a high-temperature (60 °C), the flexible base-layer asphalt material softens. The increase in loading rate causes the transverse strain that is generated by the flexible base layer to easily form accumulation, which leads to a significant increase in the transverse strain at the top and bottom of the base layer.

As shown in Figure 10b and Figure 11b, for semi-rigid base-layer asphalt pavement, at a normal temperature (30 °C), the semi-rigid base-layer material itself has a certain degree of stiffness. This can better transfer and disperse stress, and the increased load rate on the top of the base layer and the bottom of the transverse strain is minimal. At a high temperature (60 °C), the semi-rigid base-layer material produces thermal expansion, and the increase in loading rate at this time causes the shear force on the top and bottom of the base layer to increase further, which leads to a significant increase in transverse strain.

#### 3.2.3. Analysis of the Temperature and Load Rate on Vertical Strain

As shown in Figure 12 and Figure 13, under the action of a single-wheel load, the vertical strain at the top and bottom of the base layers of large-thickness flexible base and semi-rigid base asphalt pavements alternated between tensile and compressive strains, mainly compressive strains. When the test temperature and loading rate are the same, the vertical strain at the top and bottom of the semi-rigid base-layer asphalt pavement is more complicated than that of the flexible base-layer asphalt pavement.

As shown in Figure 12a and Figure 13a, for flexible base-layer asphalt pavement, at a normal temperature (30 °C), the flexible base-layer material disperses stress through its own visco-elastic deformation, and the increase in loading rate has less influence on the vertical strain at the top and bottom of the base layer. At a high temperature (60 °C), the flexible base-layer asphalt material’s modulus of elasticity and viscosity become smaller, and it is easy to produce deformation under the action of high-speed loads, which leads to a significant increase in the vertical strain at the bottom and top of the base layer.

As shown in Figure 12b and Figure 13b, for semi-rigid base-layer asphalt pavement, at a normal temperature (30 °C), the rigidity of semi-rigid base-layer material is larger, which can effectively transfer load to the lower layer of foundation, and the increase in load rate has less effect on the vertical strain at the top and bottom of the base layer. At a high temperature (60 °C), the semi-rigid base material experiences thermal expansion: the top of the base layer is constrained by the surface layer and the base layer, the base layer and the substrate constrain the bottom of the base layer, and the stress generated by the thermal expansion of the base-layer material can not be freely released [30,31,32]. When the load rate increases, the base layer is subjected to larger compressive stress in addition to the larger compressive stress from the upper load; it will also be subjected to a larger shear, which leads to a significant increase in the vertical strain of the base layer at the top and the bottom.

### 3.3. Cyclic-Wheel Load Test

A cyclic-wheel load test was carried out on ATB and CTB asphalt pavement structures at a test temperature of 30 °C. The wheel loading rate was 42 times/min, the loading intensity was 0.7 MPa, and the specimen was loaded until it was damaged or the number of cyclic wheel loads reached one million. The stress and strain data were collected at the tops and bottoms of base layers of the two types of asphalt pavements, and the fatigue performance was analyzed. Based on JTG D50-2017 [33] “Highway Asphalt Pavement Design Code”, the fatigue life of flexible base asphalt mixture (ATB) is calculated as follows:

(1) $$N_{f1}=6.32\times 10^{15.96-0.29\beta }k_{a}k_{b}k_{T1}^{-1}{\left(\frac{1}{\varepsilon_{a}}\right)}^{3.97}{\left(\frac{1}{E_{a}}\right)}^{1.58}{\left(VFA\right)}^{2.72}$$

where *ε_a_* is the tensile strain at the bottom of the asphalt mixture layer, 10^−6^; *k_T_*_1_ is the temperature adjustment factor; *VFA* is the saturation degree of asphalt mixture, %; *E_a_* is the dynamic compression modulus of asphalt mixture at 20 °C, MPa; *k_b_* is the fatigue loading mode coefficient; *k_a_* is the seasonal permafrost region adjustment factor; *β* is the target reliability index; *N*_f1_ is the number of times of the asphalt mixture number of loadings that produce fatigue cracking.

The fatigue life of the cement-stabilized mixture (CTB) for the semi-rigid base is calculated as follows:

(2) $$N_{f2}=k_{a}k_{T2}^{-1}10^{a-b\frac{\sigma_{t}}{R_{s}}+k_{c}-0.57\beta }$$

where *σ_t_* is the bottom tensile stress of the inorganic binding material stabilized layer, MPa; *β* is the target reliability index; *k_c_* is the field comprehensive correction coefficient; *a*, *b* is the regression coefficient of the fatigue test; *R_s_* is the bending and tensile strength of the inorganic stabilized binding material, MPa; *k_T_*_2_ is the temperature adjustment coefficient; *k_a_* is the adjustment coefficient of the seasonal permafrost region; *N*_f2_ is the number of loading times of the fatigue cracking produced by cement-stabilized mixtures.

**Figure 14:**
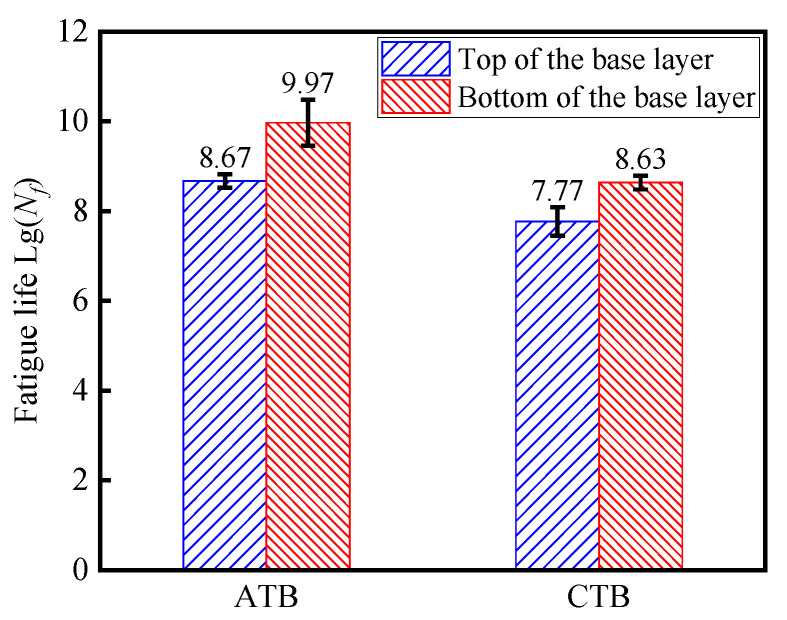
Fatigue life of asphalt pavements with different base layers.

Based on Equations (1) and (2), the fatigue life of the tops and bottoms of the base layers of two types of asphalt pavements under cyclic-wheel loading is calculated, as shown in Figure 14. Related research [5] proposed that the fatigue limit criterion for long-life (design life ≥ 40 years) asphalt pavement is that the ultimate axle load is greater than or equal to 5 million times when the strain reaches 65 με.

**Figure 15:**
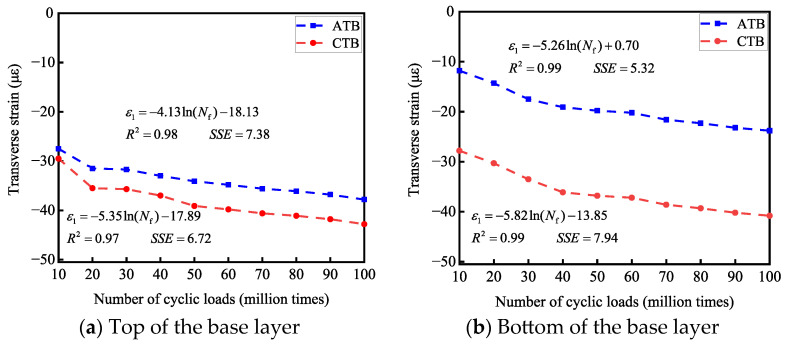
Effect of the number of cyclic loads on transverse strain.

**Figure 16:**
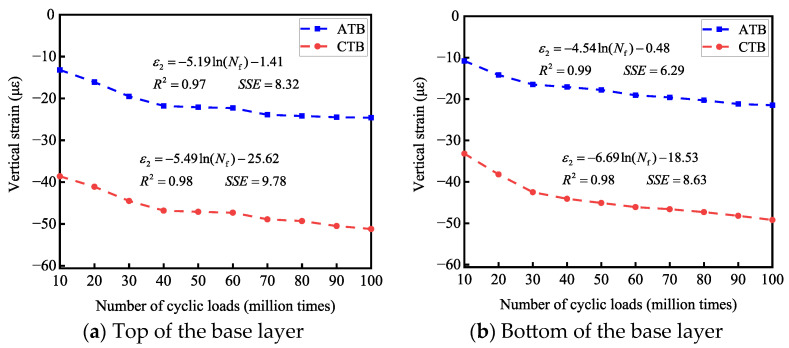
Effect of the number of cyclic loads on vertical strain.

Based on this criterion, this paper fits the transverse strains *ε*_1_ generated by the tops and bottoms of the base layers of the two types of asphalt pavement structures under cyclic loads with the number of cyclic-wheel loads *N*_f_, and analyzes the related pavement structures for long-life determination from the perspective of transverse strains. The long-life judgment analysis of the two asphalt pavement structures is shown in Figure 15; the vertical strain *ε*_2_ generated at the tops and bottoms of the base layers of the two asphalt pavement structures under cyclic loading is fitted with the number of cyclic-wheel loads *N*_f_ so as to carry out the long-life judgment analysis of the relevant pavement structures from the perspective of vertical strains, as shown in Figure 16.

#### 3.3.1. Fatigue Life Calculation

As shown in Figure 14, under the action of cyclic-wheel load, the fatigue life of the flexible base-layer asphalt pavement structure at the top and bottom of the base layer is larger than that of the semi-rigid base-layer asphalt pavement. This is due to the rigidity of the semi-rigid base layer, which is larger; in the cyclic loading effect of the base layer, it is easy to produce a sizeable tensile stress at the bottom of the base layer. When the tensile stress exceeds the limit of the tensile strength of the semi-rigid base-layer material, fatigue damage occurs at the bottom of the base layer. Compared with the semi-rigid base layer, the deformation capacity of the flexible base layer helps reduce the tensile stress transmitted from the surface layer to avoid fatigue damage. Secondly, due to the brittleness of the semi-rigid base-layer material, cracks appearing at the bottom of the semi-rigid base layer under cyclic loading will rapidly extend to the entire base and surface layers, reducing the fatigue life of the semi-rigid base-layer asphalt pavement. Finally, when small cracks appear at the bottom of the flexible base-layer asphalt pavement, the flexible base-layer material has good viscoelasticity and self-healing abilities, which can effectively slow down the expansion of cracks and prolong the fatigue life of the flexible base-layer asphalt pavement [34,35,36].

#### 3.3.2. Transverse Strain Long-Life Determination Analysis

As shown in Figure 15, combined with the fitting relationship between the transverse strain and the number of loads, when the transverse strain reaches 65 με, for flexible base-layer asphalt pavement, the number of cyclic loads at the top of the base layer is 8.6 million times, the number of cyclic loads at the bottom of the base layer is 27 million times, and the number of cyclic loads at the top and bottom of the base layer is greater than 5 million times; this is judged as long-life asphalt pavement. For semi-rigid base-layer asphalt pavement, the number of cyclic loads at the top of the base layer is 0.675 million times, the number of cyclic loads at the bottom of the base layer is 0.651 million times, and the number of cyclic loads at the top and bottom of the base layer is less than 5 million times. As such, it is determined that the semi-rigid base-layer asphalt pavement cannot satisfy the requirements of a long service life from the perspective of transverse strain.

#### 3.3.3. Vertical Strain Long-Life Determination Analysis

As shown in Figure 16, combined with the vertical strain and the number of loads, when the vertical strain reaches 65 με, for the flexible base-layer asphalt pavement, the number of cyclic loads at the top of the base layer is 21 million times, the number of cyclic loads at the bottom of the base layer is 27 million times, and the number of cyclic loads at the top and bottom of the base layer is more than 5 million times; this is judged as long-life asphalt pavement. For semi-rigid base-layer asphalt pavement, the number of cyclic loads at the top of the base layer is 0.132 million times, the number of cyclic loads at the bottom of the base layer is 1.04 million times, and the number of cyclic loads at the top and bottom of the base layer is less than 5 million times. As such, this is a semi-rigid base-layer asphalt pavement that cannot satisfy the requirements of a long service life from the perspective of vertical strain.

## 4. Conclusions and Recommendations

The mechanical response characteristics of large-thickness flexible base-layer and semi-rigid base-layer asphalt pavements in static load and single-wheel load tests are compared and analyzed from the perspectives of material properties, structural responses and force mechanisms. The fatigue performance of the two types of asphalt pavement structures under the action of cyclic-wheel loads is investigated from the perspectives of stress state, tensile stress distribution, and crack extension. Furthermore, the two types of asphalt pavement structures are analyzed in terms of determining their long-term performance. The following conclusions can be drawn from this study:

(1) In the interval of 1.3 MPa ≥ load intensity ≥ 0.5 MPa, with an increase in static load, the transverse and vertical strain generated at the top and bottom of the base layer of ATB pavement increased slowly with a slight increase; the transverse and vertical strain generated at the top of the base layer of the CTB pavement were more sensitive to heavy traffic load, and the transverse and vertical strain generated at the bottom of the base layer increased uniformly with the increase in static load.
(2) At a normal temperature (30 °C), the stress and strain at the tops and bottoms of the base layers of ATB and CTB pavements under a single-wheel load are less affected by the loading rate. At a high temperature (60 °C), the stress generated at the top and bottom of the base layer of ATB pavement gradually increased with an increase in the loading rate. The stresses generated at the top and bottom of the base layer of CTB pavement rapidly increased with the increase in loading rate, and the increase is more significant at the top of the base layer.
(3) Under the action of a single-wheel load, the transverse strain and vertical strain generated at the top and bottom of the base layer of ATB and CTB pavement alternately changed in tensile and compressive phenomena, which was mainly compressive strain, where the strain change in CTB pavement was more complicated.
(4) Compared with the CTB pavement, the ATB pavement has a more uniform stress distribution at the top of the base layer under cyclic loading. At the bottom of the base layer, it can effectively reduce the tensile stress transmitted by the surface layer, alleviate the development of cracks, and have better fatigue resistance.
(5) At a normal temperature (30 °C), when the load strength is 0.7 MPa, the large-thickness flexible base-layer asphalt pavement (38 cm) designed in this paper can meet the long service life requirements at the top and bottom of the base layer.

This article conducted laboratory experiments and analysis on thick, flexible-base asphalt pavement. Due to the differences in sample size, artificial interlayer adhesion, and laboratory environment compared to the actual service state of the pavement, the test results have certain limitations. In the future, long-term tracking and testing will be conducted on a full-size flexible-base asphalt pavement test section to verify the reliability of laboratory test results and establish a more accurate correlation model between laboratory tests and actual pavement performance.

## Figures and Tables

**Table 1:** Test results for SBS-modified asphalt properties.

Test Items	Test Results	Unit	Test Method
Penetration (25 °C, 100 g, 5 s)	52.8	0.1 mm	T 0604-2011
Softening point TR&B	85.0	°C	T 0606-2011
Ductility (5 °C, 5 cm/min)	32.2	cm	T 0605-2011
Solubility	99.3	%	T 0607-2011
Elastic recovery (25 °C)	91	%	T 0662-2000
Dynamic viscosity (135 °C)	2.3	Pa·s	T 0625-2011
	Residue after TFOT		
Quality change	−0.11	%	T 0609-2011
Penetration ratio (25 °C)	77	%	T 0604-2011
Ductility (5 °C, 5 cm/min)	15.7	cm	T 0605-2011

**Table 2:** Test results for coarse aggregate properties.

Test Items	Test Results	Unit	Test Method
Apparent relative density	2.728	-	T 0304-2005
Relative density of gross volume	2.708	-	T 0304-2005
Water absorption capacity	0.98	%	T 0307-2005
Mud content (<0.075 mm)	0. 1	%	T 0310-2005
Crushing value	13.5	%	T 0316-2000
Content of needle and flake particles	10.9	%	T 0312-2005

**Table 3:** Test results for fine aggregate properties.

Test Items	Test Results	Unit	Test Method
Angularity (flow time)	37	s	T 0345-2005
Apparent relative density	2.701	-	T 0328-2005
Mud content (<0.075 mm)	0.32	%	T 0333-2005
Sand equivalent	77.58	%	T 0334-2005
Ruggedness (>0.3 mm)	2.5	%	T 0340-2005

**Table 4:** Test results for mineral filler properties.

Test Items	Test Results	Unit	Test Method
Apparent relative density	2.691	-	T 0352-2000
Hydrophilicity coefficient	0.61	%	T 0353-2000
Appearance	No agglomeration	-	T 0353-2000
Thermal stability	No obvious discoloration	-	T 0355-2000

**Table 5:** Test results for cement properties.

Test Items	Test Results	Unit	Test Method
Fineness	1.7	%	T 0502-2005
Initial condensation time	218	min	T 0505-2020
Final condensation time	316	min	T 0505-2020
Stability	Qualified	-	T 0505-2020

**Table 6:** Test results for ATB-25 properties.

ATB-25	VV	VMA	VFA	Stability	Flow Value	Asphalt–Aggregate Ratio
Unit	%	%	%	kN	mm	%
Test results	4.9	13.2	66.0	10.2	3.5	3.6

**Table 7:** Test results for CTB-25 properties.

CTB-25	Maximum Dry Density	Cement Content	7-Day Unconfined Compressive Strength
Unit	g/cm^3^	%	MPa
Test results	2.18	5.0	5.9

## Data Availability

The original contributions presented in this study are included in the article. Further inquiries can be directed to the corresponding authors.
